# *Cordyceps militaris* Immunomodulatory Protein Promotes the Phagocytic Ability of Macrophages through the TLR4-NF-κB Pathway

**DOI:** 10.3390/ijms222212188

**Published:** 2021-11-11

**Authors:** Hong-Bo Fan, Yuan Zou, Qing Han, Qian-Wang Zheng, Ying-Li Liu, Li-Qiong Guo, Jun-Fang Lin

**Affiliations:** 1 Department of Bioengineering, College of Food Science, South China Agricultural University, 483 Wushan Road, Guangzhou 510640, China; fanhb@stu.scau.edu.cn (H.-B.F.); zouyuan@scau.edu.cn (Y.Z.); zhqianw@scau.edu.cn (Q.-W.Z.); 2 Research Center for Micro-Ecological Agent Engineering and Technology of Guangdong Province, Guangzhou 510640, China; 3 College of Marine Science, South China Agricultural University, 483 Wushan Road, Guangzhou 510640, China; hq2021@yeah.net; 4 Beijing Advanced Innovation Center for Food Nutrition and Human Health, Beijing Technology and Business University, Beijing 100048, China; liuyingli@th.btbu.edu.cn

**Keywords:** *Cordyceps militaris*, immunomodulatory protein, macrophage, phagocytosis, immunity

## Abstract

Enhancing the phagocytosis of immune cells with medicines provides benefits to the physiological balance by removing foreign pathogens and apoptotic cells. The fungal immunomodulatory protein (FIP) possessing various immunopotentiation functions may be a good candidate for such drugs. However, the effect and mechanism of FIP on the phagocytic activity is limitedly investigated. Therefore, the present study determined effects of *Cordyceps militaris* immunomodulatory protein (CMIMP), a novel FIP reported to induce cytokines secretion, on the phagocytosis using three different types of models, including microsphere, *Escherichia Coli* and *Candida albicans*. CMIMP not only significantly improved the phagocytic ability (*p* < 0.05), but also enhanced the bactericidal activity (*p* < 0.05). Meanwhile, the cell size, especially the cytoplasm size, was markedly increased by CMIMP (*p* < 0.01), accompanied by an increase in the F-actin expression (*p* < 0.001). Further experiments displayed that CMIMP-induced phagocytosis, cell size and F-actin expression were alleviated by the specific inhibitor of TLR4 (*p* < 0.05). Similar results were observed in the treatment with the inhibitor of the NF-κB pathway (*p* < 0.05). In conclusion, it could be speculated that CMIMP promoted the phagocytic ability of macrophages through increasing F-actin expression and cell size in a TLR4-NF-κB pathway dependent way.

## 1. Introduction

Edible fungi, especially mushrooms, provide various kinds of immunomodulatory proteins, including lectins, ribosome inactivating proteins, ribonucleases, laccases and fungal immunomodulatory protein (FIP) [1]. These proteins can regulate the mitosis, proliferation, differentiation and maturation of immune cells, resulting in strengthening the immunity [2,3]. Among these immunomodulatory proteins, FIP accounts for the highest proportion and has been most extensively investigated. The first reported FIP, named Ling Zhi-8 (LZ-8 or FIP-glu), was revealed to hemagglutinate sheep red blood cells and reduce systemic anaphylaxis reactions in mice in 1989 [4]. Until now, twenty-two FIPs sharing high amino acid sequence and protein structure similarity have been identified [5]. Meanwhile, new immunological activities of these FIPs are revealed, covering anti-tumor, promotion of lymphocyte proliferation, antiviral, antimicrobial and induction of cytokines expression [2,5]. Future studies on FIPs focus not only on the identification of new FIPs, but also on expanding the functional activity of these known FIPs, such as improving the phagocytic ability of immune cells.

Phagocytosis is a form of host defense mainly performed by cells of myeloid origin, including monocytes, macrophages and polymorphonuclear neutrophils. In the process of phagocytosis, foreign pathogens and insoluble large particles are ingested with the help of actin polymerization [6]. Upon ingestion, microbes in the phagolysosome are killed and degraded by microbicidal enzymes of the lysosome. In the context of immunity, phagocytosis is not only an essential function of innate immunity but also a promotor of the initiation and development of adaptive immune responses [7]. In mammals, phagocytosis plays vital roles in the physiological processes of tissue remodeling, nutrition, inflammation and immunity [8]. Therefore, maintaining or promoting the phagocytic ability of immune cells is of great importance which, at the same time, makes immunomodulatory drugs and adjuvants a necessity for keeping human health.

The phagocytosis-regulatory function of fungi immunomodulatory proteins has been preliminarily measured. The lectin of oyster mushroom (*Pleurotus florida*) possesses the ability of protecting the phagocytic activity of arsenic-induced hepatocytes [9]. In addition, the lectin from *Latiporus sulphureus* greatly improves the phagocytic activity of murine macrophages [10]. Remarkably, the immunomodulatory protein of *Antrodia camphorate* (ACA) enhances the phagocytic activity of murine peritoneal macrophages [11]. Hence, fungi proteins possessing the phagocytosis-regulatory function may be good candidates for agents maintaining human phagocytic activity due to the natural and functional advantages. However, the regulatory mechanism of these proteins on phagocytosis has not been revealed. Additionally, apart from ACA, the effect of other FIPs on the phagocytosis of immune cells is unclear. Therefore, it is necessary to explore the influence and underlying mechanism of FIP on the phagocytic function of immune cells.

*Cordyceps militaris* is a well-known fungus used in traditional Chinese medicine that provides various health benefits and has been classified as a drug in the Chinese Pharmacopoeia [12,13,14]. Proteins of *C. militaris* possess multiple pharmacological activities including antifungal, anticancer, antivirus and mitogenic [15,16,17,18]. In our previous study, we identified a novel FIP, named *Cordyceps militaris* immunomodulatory protein (CMIMP). CMIMP belongs to the new branch of the FIP family and possesses the ability of inducing proinflammation cytokines secretion [19]. In the current study, we determined the effect of CMIMP on phagocytosis using three types of models containing fluorescent beads, green fluorescent protein (GFP)-expressing *Escherichia Coli* and *Candida albicans*. Furthermore, the modulatory mechanism was revealed as well. This research expands the immunological function of FIP and provides a reference for the study on physiological activity of bioactive compounds.

## 2. Results and Discussion

### 2.1. CMIMP Enhanced Phagocytic and Bactericidal Activity of Macrophages

Phagocytosis plays a vital role in recognizing and presenting antigens to members of the immune system, resulting in eliminating invading pathogens, tumor cells and apoptotic cells from organs [20]. Phagocytosis can be augmented by compounds of edible fungi such as polysaccharides and lectins [11,21,22]. However, there are limited studies about the effect of FIP on the phagocytic activity of immune cells. Taking these aspects into consideration, we intended to determine the effect of FIP on the phagocytosis of macrophages and reveal the underlying mechanism using CMIMP as a model.

**Figure 1:**
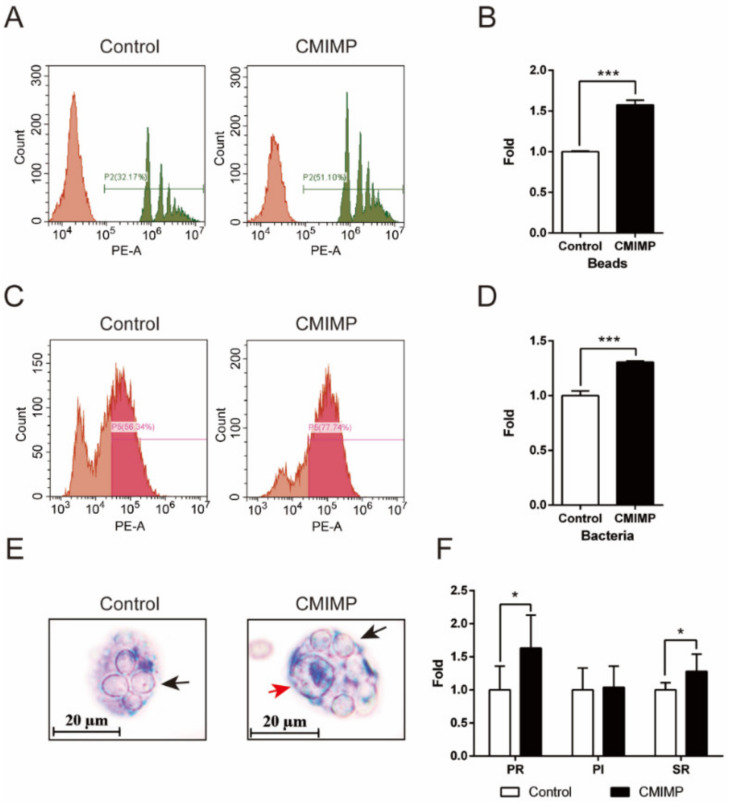
Effect of CMIMP on the phagocytic ability of RAW264.7 macrophages. (**A**,**B**) Effect of CMIMP (80 ng/mL) on the phagocytosis to fluorescent beads. Percentage of phagocytic macrophages was determined by the flow cytometry. (**C**,**D**) Effect of CMIMP (80 ng/mL) on the phagocytosis to GFP-expressed *E. Coli*. Percentage of phagocytic cells was determined by the flow cytometry. (**E**,**F**) Effect of CMIMP (80 ng/mL) on the phagocytosis to *Candida Albicans*. Cells were stained with methylene blue and imaged. The red arrow points to the nucleus and black arrow points to phagocytized *Candida Albicans*. PR, PI and SR were counted (n = 30). The data was analyzed by GraphPad Prism 6 software. Difference with *p* < 0.05 (*) and *p* < 0.001 (***) were considered statistically significant.

In this study, the phagocytosis of RAW264.7 macrophages to beads, bacteria and *Candida albicans* was firstly measured. As shown, compared with the Control group, the percentage of macrophages phagocytizing beads in the CMIMP group was highly improved (*p* < 0.001) (Figure 1A,B). As to *E. coli*, similar results were observed in Figure 1C,D (*p* < 0.001). These indicated that the phagocytic activity of macrophages was increased by CMIMP. Remarkably, for *Candida albicans*, not only the PR, but also the SR was markedly enhanced by CMIMP (*p* < 0.05) (Figure 1E,F), suggesting that the bactericidal ability of macrophages was augmented as well. Similar to CMIMP, the immunomodulatory protein from *Antrodia camphorate* (ACA), either native or recombinant, possesses the ability to promote the phagocytic activity of murine peritoneal macrophages [11]. Since ACA and CMIMP share the conserved cerato-platanin domain (Pfam no. 07249), it can be speculated that fungi immunomodulatory proteins with the same domain may possess the ability of increasing the phagocytic and bacterial activity of macrophages, resulting in enhancing the macrophage-mediated immune response.

### 2.2. CMIMP Enlarged the Size and F-Actin Expression of Macrophages

**Figure 2:**
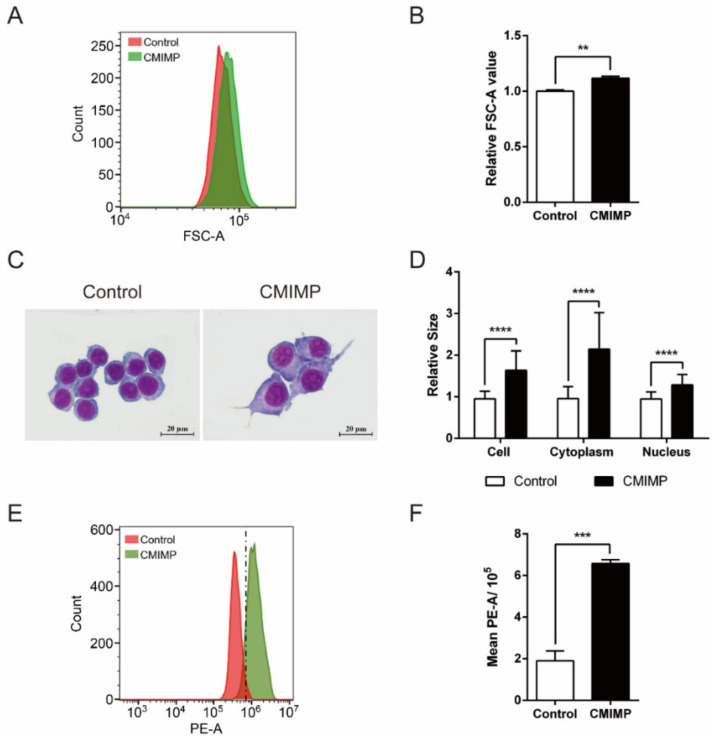
Effect of CMIMP on cell size and F-actin expression of RAW264.7 macrophages. (**A**,**B**) Effect of CMIMP (80 ng/mL) on FSC-A of macrophages. After treatment, cells were harvested and subjected to the flow cytometry to measure the FSC. (**C**,**D**) Effect of CMIMP (80 ng/mL) on the relative size of cell, cytoplasm and nucleus. Cells were stained by Giemsa staining and the size was analyzed by FlowJo (n = 30). (**E**,**F**) Effect of CMIMP (80 ng/mL) on F-actin expression. F-actin was labeled with FITC-phalloidin and measured by flow cytometry. The data was analyzed by GraphPad Prism 6. Difference with *p* < 0.01 (**), *p* < 0.001 (***) and *p* < 0.0001 (****) were considered statistically significant.

For granulocyte, the phagocytic ability to particles is directly proportional to the initial cell size [23]. Therefore, the size of RAW264.7 macrophages was measured to explore the mechanism of CMIMP-enhanced phagocytosis. The size was measured by the flow cytometric method and Giemsa staining. As shown in Figure 2A,B, the relative FSC-A value of macrophages in CMIMP group was greater than that of Control group (*p* < 0.01). Moreover, the Giemsa staining result displayed macrophages that became larger in volume and stretched out obvious pseudopods after treatment with CMIMP (Figure 2C). Statistical analysis exhibited that not only the area of cell, but also the area of cytoplasm and nucleus were significantly increased by CMIMP (*p* < 0.0001) (Figure 2D). These results jointly demonstrated that CMIMP could enlarge the cell size of macrophages. In accordance with our results, the increased cell size and cytoplasmic spreading occur with macrophage activation [24]. Therefore, it can be inferred that the enhanced phagocytosis may be due to the larger initial cell size and cytoskeletal rearrangement induced by CMIMP.

F-actin is an essential element of the cytoskeleton in non-muscle cells and its content correlates with the restructuring of cytoskeleton and the resultant motile behaviors, including cytokinesis, secretion and endocytosis [25]. Hence, the effect of CMIMP on F-actin expression of RAW264.7 macrophages was subsequently measured. As shown, the content of F-actin rose after CMIMP addition (*p* < 0.001) (Figure 2E,F). Comparing with the undifferentiated macrophages, the content and microfilament spreading area of F-actin is increased in M1 cells [26]. In line with that, macrophages were induced into M1 phenotype by CMIMP [19]. Taken together, these results discovered that CMIMP enhanced the expression of F-actin required for enlarging cell size.

### 2.3. Role of TLR4 in CMIMP-Mediated Cell Phagocytosis

**Figure 3:**
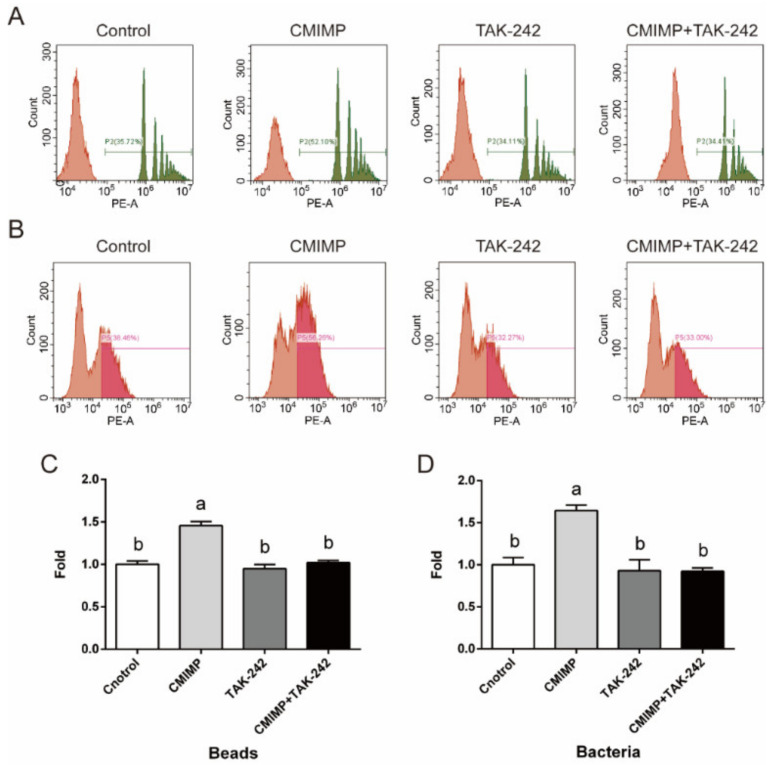
The role of TLR4 in CMIMP-enhanced phagocytosis of RAW264.7 macrophages. (**A**,**C**) Effect of TAK-242 (inhibitor of TLR4) on CMIMP (80 ng/mL) enhanced phagocytosis to fluorescent beads. Percentage of phagocytic macrophages was determined by the flow cytometry. (**B**,**D**) Effect of TAK-242 on CMIMP (80 ng/mL) enhanced phagocytosis to GFP-expressed *E. Coli*. Percentage of phagocytic cells was determined by the flow cytometry. The data was analyzed by GraphPad Prism 6 software. Bars without the same letter indicates significant difference (*p* < 0.05).

Toll-like receptors (TLRs) localizing on the cell surface are in charge of recognizing extracellular pathogens and microbial products. After ligand binding, TLRs regulate the phagocytosis process at multiple steps, including internalization and phagosome maturation [27]. In the absence of TLRs signaling, phagocytosis of bacteria is blocked [28]. In the previous study, TLR4 is verified to modulate the cytokine-inducing activity of CMIMP, while its role in CMIMP-enhanced phagocytosis is inexplicit [19]. Thus, the specific inhibitor of TLR4 (TAK-242) was used to determine whether TLR4 participated in the phagocytosis of RAW264.7 macrophages. Results displayed that CMIMP-induced phagocytosis to beads was obviously inhibited by TAK-242 (*p* < 0.05), with no effect when singly applied (Figure 3A,C). As to bacteria, similar results were observed (Figure 3B,D). These suggested that TLR4 was in charge of CMIMP-enhanced phagocytosis. Confirming that activation of TLR4 induced by LPS promotes phagocytosis as well [29].

**Figure 4:**
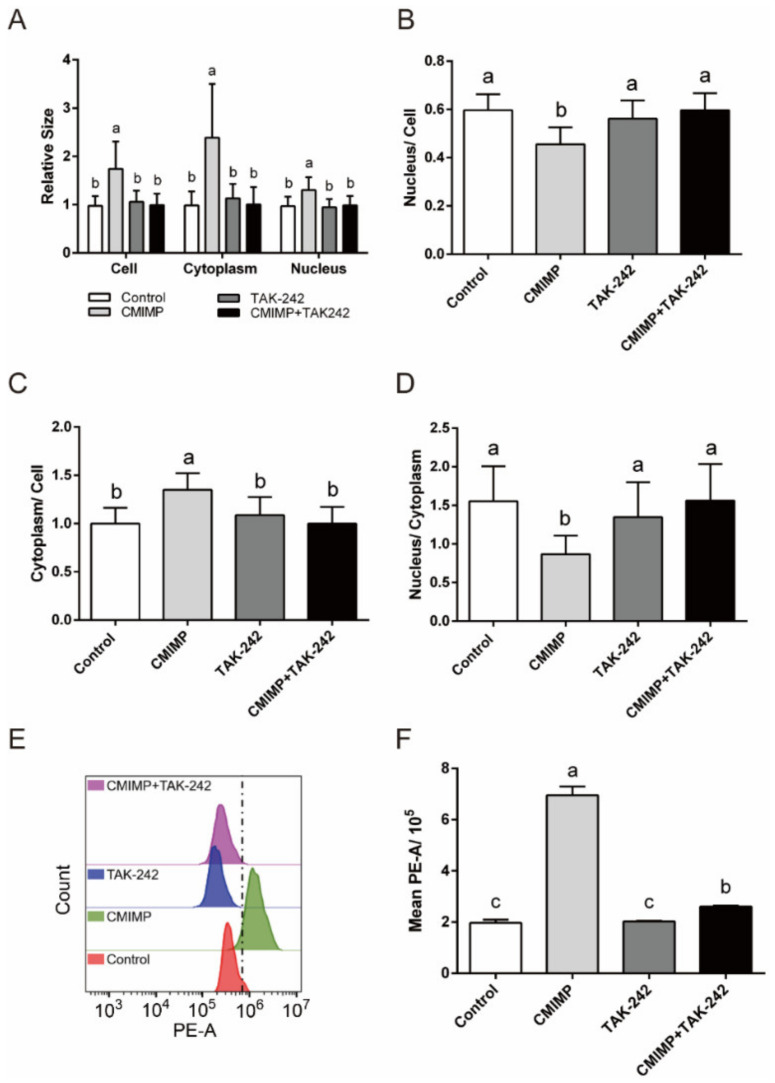
The role of TLR4 in CMIMP-enlarged cell size of RAW264.7 macrophages. (**A**) Effect of TAK-242 (inhibitor of TLR4) on CMIMP (80 ng/mL) enlarged size of cell, cytoplasm and nucleus. Cells were stained by Giemsa staining and the size was analyzed by FlowJo (n = 30). (**B**–**D**) Effect of TAK-242 on CMIMP (80 ng/mL) induced relative size change of cytoplasm and nucleus. (**E**,**F**) Effect of TAK-242 on CMIMP (80 ng/mL) increased F-actin expression. F-actin was labeled with FITC-phalloidin and measured by flow cytometry. Figure 4E was made with one replicate of each group. The data was analyzed by GraphPad Prism 6. Bars without the same letter indicates significant difference (*p* < 0.05).

In addition, the role of TLR4 in regulating cell size and F-actin expression of RAW264.7 macrophages was detected. As shown in Figure 4A, the relative size of cell, cytoplasm and nucleus in the CMIMP + TAK-242 group was significantly lower than the CMIMP group (*p* < 0.05), indicating that TLR4 could regulate the size of cell, cytoplasm and nucleus. Furthermore, compared with the Control group, the relative size of nucleus to cell markedly declined in the CMIMP group (*p* < 0.05), but this decrease was relieved in the CMIMP + TAK-242 group (Figure 4B). In contrast, compared with the Control group, the relative size of cytoplasm to cell markedly rose in the CMIMP group (*p* < 0.05), but this increase was weakened in the CMIMP + TAK-242 group (Figure 4C). The relative size of nucleus to cytoplasm was distinctly lessened by CMIMP (*p* < 0.05), but was moderated with TAK-242 applied jointly (Figure 4D). In aggregate, these indicated that TLR4 could regulate the size of cell, cytoplasm and nucleus. In agreement with our research, similar results were observed in macrophages treated with the agonist of TLR4, resulting from the increased plasticity of cytoskeleton [30]. Hence, it could be inferred that the cell size, especially the cytoplasm size, enlarged by CMIMP, was due to the elevated fluidity of cell membrane modulated by the TLR4.

For the content of F-actin, the CMIMP + TAK-242 group was significantly lower than the CMIMP group (*p* < 0.05), indicating that CMIMP-enhanced expression of F-actin was dependent on TLR4 (Figure 4E,F). Similarly, LPS, the agonist of TLR4, raises the expression of F-actin in macrophages [30]. Accordingly, these results revealed that TLR4 could enlarge cell size through increasing F-actin expression, which might answer for CMIMP-induced cell phagocytosis.

### 2.4. Role of the NF-κB Pathway in CMIMP-Mediated Cell Phagocytosis

**Figure 5:**
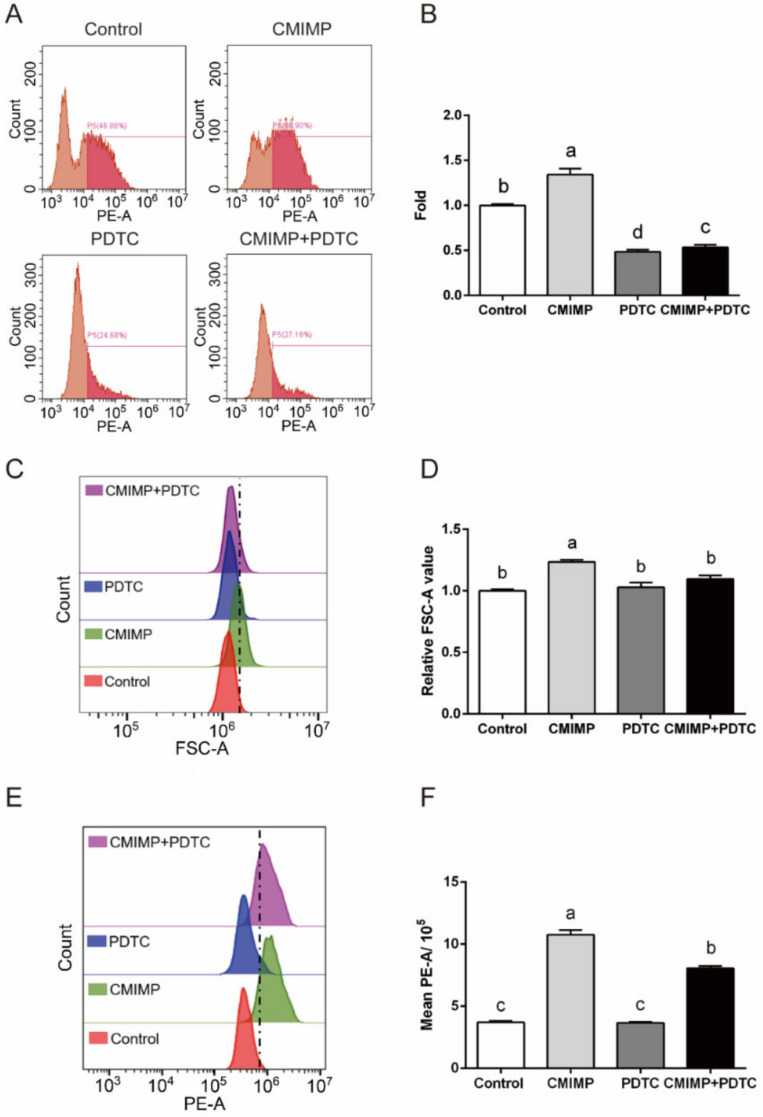
The role of NF-κB pathway in CMIMP-enhanced phagocytosis, cell size and F-actin content of RAW264.7 macrophages. (**A**,**B**) Effect of PDTC (inhibitor of NF-κB pathway) on CMIMP (80 ng/mL) enhanced phagocytosis to GFP-expressed *E. Coli*. Percentage of phagocytic cells was determined by the flow cytometry. (**C**,**D**) Effect of PDTC on CMIMP (80 ng/mL) enhanced cell size. (**E**,**F**) After treating, cells were incubated with FITC-labeled phalloidin. The fluorescence intensity was measured by flow cytometry. The data was analyzed by GraphPad Prism 6 software. Bars without the same letter indicates significant difference (*p* < 0.05).

The NF-κB pathway locates at the downstream of TLR4 and the signaling from TLR4 activates nucleus translocation of NF-κB [28]. In the previous study, the NF-κB pathway was proved to be in charge of CMIMP-induced proinflammation cytokines releasing [19]. While other pathways, such as the mitogen-activated protein kinase (MAPK) pathway, also can transmit signals from TLR4 [31]. In addition, the role of the NF-κB pathway in CMIMP-mediated phagocytosis is unknown. Thus, the specific inhibitor of the NF-κB pathway (PDTC) was used to determine whether the NF-κB pathway modulated cell phagocytosis of RAW264.7 macrophages. As shown in Figure 5A,B, compared with the Control group, the percentage of macrophages phagocytizing bacteria in the PDTC group was markedly decreased (*p* < 0.05), meaning that the NF-κB pathway took charge of the phagocytosis to *E. coli*. As to the CMIMP + PDTC group, the percentage of macrophages phagocytizing bacteria was evidently lower than the CMIMP group (*p* < 0.05), and even than the Control group (*p* < 0.05), suggesting that CMIMP-induced phagocytosis was regulated by the NF-κB pathway. A similar result was obtained in the study on phagocytosis to colonial ascidian *Botryllus schlosseri* [32]. This phenomenon might be because PDTC shows inhibition effect on the rearrangement of F-actin, which may consequently hinder the phagocytosis to pathogens [33].

Furthermore, the effect of PDTC on cell size and F-actin of RAW264.7 macrophages was tested. Figure 5C,D shows that the relative FSC-A value of the CMIMP + PDTC group was markedly lower than that of the CMIMP group (*p* < 0.05), with no significant difference between the Control group and PDTC group, indicating that CMIMP-increased cell size was relieved by PDTC. This evidenced that the NF-κB pathway regulated the size of cells as well and was in line with the results of TAK-242. For the F-actin expression, the mean PE-A value of the Control group was not different to that of the PDTC group. However, the value of the CMIMP + PDTC group was obviously lower than the CMIMP group (*p* < 0.05) (Figure 5E,F). This signified that PDTC reserved CMIMP-induced F-actin expression but showed no effect when singly used. This might be the result that PDTC limited NF-κB release and binding to DNA, which reduced the level of G-actin [33,34]. Summing up, these disclosed that CMIMP-triggered cell size and F-actin expression was dependent on the NF-κB pathway.

**Figure 6:**
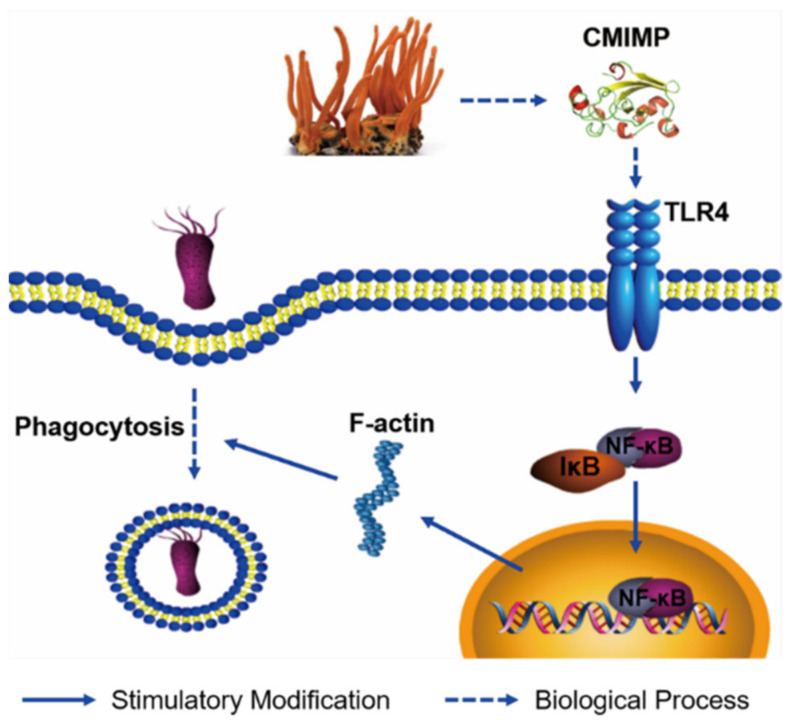
Schematic diagram shows that *Cordyceps militaris* immunomodulatory protein CMIMP enhances the expression and rearrangement of F-actin by activating the TLR4-NF-κB pathway, which consequently promotes the phagocytic ability of RAW264.7 macrophages.

Combining all results above, it can be speculated that CMIMP derived from *Cordyceps militaris* binds the membrane TLR4 of RAW264.7 macrophages, which subsequently activates the NF-κB pathway and induces NF-κB to translocate into the nucleus. Then, NF-κB promotes the expression of F-actin, leading to cell volume increase and morphological change. As a result, the phagocytosis percentage and ability of these activated cells are promoted and so is the bacterial activity (Figure 6).

## 3. Materials and Methods

### 3.1. Materials

The immunomodulatory protein CMIMP was produced by the pET32a expression system as described in our previous research [19]. Green florescent bead was purchased from Thermo Fisher Scientific (FluoSpheres F8803, Portsmouth, NH, USA). The GFP-expressed *E. coli* and *Candida albicans* were preserved in our lab. The Giemsa stain solution (G1015) and methylene blue solution (G1300) were purchased from Solarbio (Beijing, China). Fetal bovine serum was purchased from Gibco (Rockville, MD, USA) and DMEM (RXR20165) was from Quanzhou Ruixin Biological Technology Co., Ltd. (Quanzhou, Fujian, China). The specific inhibitor of TLR4 (TAK-242) and NF-κB pathway (PDTC) was obtained from MCE (Monmouth Junction, NJ, USA).

### 3.2. Measurement of the Phagocytic Activity

To detect the phagocytic activity of macrophages, the cell line RAW264.7 obtained from the Cell Resource Center of Peking Union Medical College Hospital (Beijing, China) was used as model. Cells were maintained in DMEM containing 10% fetal bovine serum, penicillin (100 unit/mL) and streptomycin (100 unit/mL) at 37 °C in a 5% CO_2_ humidified atmosphere.

To determine the phagocytic ability, the measuring strategy from Parra et al. was applied [6]. Briefly, 2 × 10^5^ cells were seeded in the 6-well plates. After treatment with CMIMP (80 ng/mL) for 24 h, cells were incubated with 1 μm florescent beads at a cell:bead ratio of 1:10 or with GFP-expressed *E. coli* (8 × 10^6^ cfu/mL) for 3 h. Then, non-ingested beads or bacteria were removed by washing with PBS supplemented with 3% BSA (Beyotime, Shanghai, China) and 4.5% D-glucose (Sigma, St. Louis, MO, USA). After trypsinization, cells were measured with CytoFLEX S (Beckman, Brea, CA, USA). Cells with no treatment and incubation were set as blank control. Percentage of cells with higher signal intensity than the blank control group was recorded and analyzed.

For the antimicrobial activity, *Candida albicans* was used as model. Briefly, after seeding and treatment, *Candida albicans* was added at a ratio of 1:5. After incubation of 60 min, 0.2% methylene blue was used for staining, observation and counting, with the inactive blue and the active transparency. The phagocytosis ration (PR, the percentage of phagocytic cells), phagocytic index (PI, the number of phagocytized fungi per cell) and sterilizing rate (SR, ratio of the sterilized fungi to the total engulfed fungi) was statistically analyzed.

### 3.3. Measurement of Cell Size

To measure cell size, the flow cytometry and Giemsa staining were applied. For the flow cytometric method, cells (2 × 10^5^ cells/mL) were firstly seeded in 6-well plates and treated with CMIMP (80 ng/mL). After washing with PBS and digesting with trypsin (GIBCO), cells were subjected to flow cytometry to measure the forward scatter (FSC). The raw data was analyzed by FlowJo software (BD, Gaithersburg, MD, USA).

As to the Giemsa staining method, cells were seeded on the glass slide. After treatment with CMIMP, cells were fixed with methanol and then stained with Giemsa stain (Solarbio, Beijing, China) for 30 min. After washing with PBS, the slide was observed and imaged using a Carl Zeiss Axio Observer A1 Microscope (Oberkochen, Germany). The size of cell and nucleus were calculated by measuring cell diameters in four randomly selected fields using ImageJ2 (https://imagej.net/software/imagej2/, Accessed on 15 October 2021). The size of cytoplasm was obtained by subtracting nucleus size from cell size.

### 3.4. Measurement of F-Actin Expression

F-actin expression was measured with FITC-labeled phalloidin (Solarbio). After seeding and treatment, cells were incubated with phalloidin for 10 min, accompanied with 4% (*w*/*v*) paraformaldehyde and 1% (*w*/*v*) saponin. Then, cells were washed with PBS, harvested with cell scarper and determined with CytoFLEX S. The mean PE value was collected and analyzed.

### 3.5. Assessing the Role of TLR4-NF-κB Pathway in Cell Phagocytosis

To assess the role of TLR4-NF-κB pathway in CMIMP-mediated phagocytosis, specific inhibitor of TLR4 (TAK-242, 20 nM, MCE, Monmouth Junction, NJ, USA) and NF-κB pathway (PDTC, 30 μM, MCE) were used to treat cells alone or combined with CMIMP. After seeding and treatment, the phagocytic activity, cell size and F-actin expression were determined using the method mentioned forehead.

### 3.6. Statistical Analysis

Experiments were conducted in triplicate and the data were expressed as means ± SD (n = 6). Statistical significance was determined by one-way ANOVA (Student’s test) using GraphPad Prism 6 software (San Diego, CA, USA). Difference with *p* < 0.05 (*), *p* < 0.01 (**), *p* < 0.001 (***) and *p* < 0.0001 (****) were considered statistically significant.

## 4. Conclusions

In summary, CMIMP enhanced the expression and rearrangement of F-actin by activating the TLR4-NF-κB pathway which consequently promoted the phagocytic ability of macrophages. This study elucidates the effect and mechanism of CMIMP on the phagocytosis, which not only demonstrates that CMIMP is an excellent candidate for immunomodulatory agents, but also provides a reference for the study on phagocytic modulation of bioactive compounds.

## Data Availability

The data presented in this study are available on request from the corresponding author.
